# Gene model for the ortholog of *sad* in *Drosophila cardini*

**DOI:** 10.17912/micropub.biology.002229

**Published:** 2026-06-25

**Authors:** Payal Patel, Pablo Chialvo, Clare Scott Chialvo

**Affiliations:** 1 Biology, Appalachian State University, Boone, North Carolina USA

## Abstract

We developed a gene model for the *shadow* ortholog ( *sad* ) in the ASM1890373v1 Genome Assembly (GenBank Accession: GCA_018903735.1) of *Drosophila cardini* . This ortholog was characterized as part of a developing dataset for a comparative study of detoxification gene family evolution in the *immigrans* - *tripunctata* radiation of the genus *Drosophila* using an adapted Genomics Education Partnership gene annotation protocol for Course-based Undergraduate Research Experiences.

**Figure 1. Genomic neighborhood and gene model for sad in D. cardini:** (A) **Synteny comparison of the genomic neighborhoods for *sad* in *Drosophila melanogaster* and *D. cardini* .** Thin underlying arrows indicate which DNA strand the target gene, *sad* , is located on in *D. melanogaster* (top) and *D. cardini* (bottom). The thin arrow pointing to the right indicates that *sad* is on the positive strand in *D. melanogaster* , and the thin arrow pointing to the left indicates that *sad* is on the negative strand in *D. cardini* . The wide gene arrows pointing in the same direction as *sad* are on the same strand relative to the thin underlying arrows, while wide gene arrows pointing in the opposite direction of *sad* are on the opposite strand relative to the thin underlying arrows. White gene arrows in *D. cardini* indicate orthology to the corresponding gene in *D. melanogaster* , while black gene arrows indicate non-orthology. Gene symbols given in the *D. cardini* gene arrows indicate the orthologous gene in *D. melanogaster* , while the locus identifiers are specific to *D. cardini* . (B) **Gene Model in GEP UCSC Track Data Hub** (Raney et al., 2014). The coding-regions of *sad* in *D. cardini* are displayed in the User Supplied Track (red); coding sequences (CDS) are depicted by thick rectangles and introns by thin lines with arrows indicating the direction of transcription. Subsequent evidence tracks include Spaln of *D. melanogaster* Proteins (purple, alignment of Ref-Seq proteins from *D. melanogaster* ), Coding Regions Predicted by Augustus (blue), GeMoMa (teal), and NSCAN PASA-EST (dark green), and RNA-Seq from mixed sex adult flies (brown; alignment of Illumina RNA-Seq reads from *D. cardini* – Erlenbach et al. 2023). (C) **Dot Plot of sad-PA in *D. melanogaster* ( *x* -axis) vs. the orthologous peptide in *D. cardini* ( *y* -axis).** Amino acid number is indicated along the left and bottom; CDS number is indicated along the top and right, and CDSs are also highlighted with alternating colors. Line breaks in the dot plot indicate areas of with low amino acid sequence identity and/or gaps between species. In CDS 1, there is a long break (purple box – a), and in CDS 3 there is a shorter break (light blue box – b). (D) **Idiosyncrasies in protein alignment.** CDS 1 contains one long break in the protein alignment and CDS 3 contains a shorter break in the protein alignment that indicate low levels of sequence similarity. In the CDS 1 break (purple box – a), two gaps were added to the *D. cardini* sequence to align it with the *D. melanogaster* sequence, and there are areas where the sequence includes several amino acids that are chemically dissimilar. The CDS 3 break (light blue box – b), includes the addition of two gaps to the *D. cardini* sequence and several dissimilar amino acids.

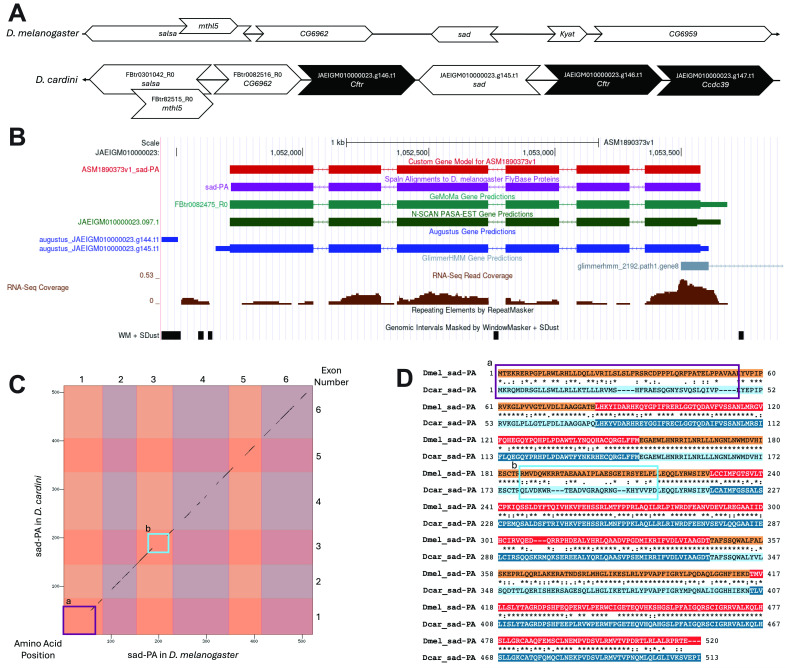



## Description

***Introduction***

*This article reports a predicted gene model generated by undergraduate work using a structured gene model annotation protocol defined by the Genomics Education Partnership (GEP; thegep.org ) for Course-based Undergraduate Research Experience (CURE). The following information in this box may be repeated in other articles submitted by participants using the same GEP CURE protocol for annotating Drosophila species orthologs of Drosophila melanogaster detoxification genes.*

“Within insects, the process of detoxifying xenobiotics and host secondary metabolites is a three-phase process that involves functionalization, conjugation, and excretion of these compounds. Expansions of known detoxification gene families ( *e.g.* , cytochrome P450s) is associated with diet breadth and insecticide resistance (Ranson et al., 2002; Després et al., 2007; Rane et al., 2016). With the increasing availability of high-quality genomes for non-model organisms, including *Drosophila* species beyond *D. melanogaster* , it is now possible to perform large scale comparative studies (Robinson et al., 2011; Kim et al., 2021; Threfall and Baxter, 2021). Careful manual annotation and curation of gene models can improve upon computational gene predictions in non-model species, which aids the accuracy of studies on gene and genome evolution (Mudge and Harrow, 2016; Tello-Ruiz et al., 2019). To aid in these annotations, the Genomics Education Partnership (thegep.org) developed a curriculum involving web-based tools that allow undergraduates to engage in authentic course-based research focused on manually annotating genes in non-model species (Rele et al., 2023). The orthologous gene models, including the one presented here, then provide a reliable basis for further evolutionary genomic analyses when made available to the scientific community. The gene ortholog described here in *D. cardini* for *shadow* ( *sad* ), a member of the cytochrome P450 monooxygenase gene family, was characterized as part of a developing dataset for a comparative study of detoxification gene families in the *immigrans* - *tripunctata* radiation of the genus *Drosophila* .” (Williams et al., 2026)

In the subgenus *Drosophila* , *D. cardini* Sturtevant 1916 is a member of the *cardini* subgroup in the *cardini* species group of the *immigrans-tripunctata* radiation (Heed and Krishnamurthy, 1959; Bächli, 2005). Species in the *cardini* subgroup are found in the mainland Neotropics, and the range of *D. cardini* extends from Florida to Brazil (Heed, 1962). Members of the *cardini* group primarily feed and develop on fruit and flowers (Markow and O'Grady, 2008). However, *D. cardini* is also reported to feed on mushrooms and can tolerate the cyclopeptide toxin α-amanitin (Stump et al., 2011).

Cytochrome P450 monooxygenases (CYPs) are a family of phase I detoxification enzymes that are found in almost all aerobic organisms and act by oxidizing compounds to make them more polar (Stegeman and Livingstone, 1998; Li et al., 2007). The enzymes in this family vary in both their substrate specificity and the range of metabolites that they produce (Rendic and Di Carlo, 1997; Scott, 1999). Furthermore, the substrate specificity of CYPs can be altered by a change in a single amino acid (Lindberg and Negishi, 1989). A member of the Halloween gene group, *shadow* ( *sad* ) is a mitochondrial CYP whose expression is restricted to the prothoracic gland cells of the ring gland (Gilbert, 2004). Halloween genes act in the biosynthetic pathway that produces 20-Hydroxyecdysone, a hormone that controls metamorphosis and development in arthropods (Gilbert and Warren, 2005; Rewitz et al., 2006). *sad* mutants exhibit phenotypes similar to *disembodied* mutants and die prior to completing embryogenesis displaying abnormal cuticular development (Warren et al., 2002; Gilbert ,2004).

We propose a gene model for the *D. cardini* ortholog of the *D. melanogaster* *shadow* ( *sad* ) gene. The genomic region of the ortholog corresponds to the Augustus gene prediction JAEIGM010000023.g145.t1 in the ASM1890373v1 Genome Assembly of *D. cardini* (GCA_018903735.1 – Kim et al., 2021). This model is based on RNA-Seq data from *D. cardini* (Erlenbach et al. 2023; https://doi.org/10.5061/dryad.hdr7sqvq2 ) and *sad* in *D. melanogaster* using FlyBase release FB2024_02 (GCA_000001215.4; Gramates et al., 2022; Jenkins et al., 2022; Larkin et al., 2021).

***Synteny -*** The reference gene, *sad,* occurs on chromosome 3R in *D. melanogaster* and is flanked upstream by *CG6959* and *Kynurenine aminotransferase* ( *Kyat* ) and downstream by *CG6962* , *salsa* ( *salsa* ), and *methuselah-like 5* ( *mthl5* ) which is nested in *salsa* . The *tblastn* search of *D. melanogaster* sad-PA (query) against the *D. cardini* (GenBank Accession: GCA_018903735.1 Genome Assembly (database) placed the putative ortholog of *sad* within contig_901 (JAEIGM010000023) which corresponds to Augustus gene prediction JAEIGM010000023.g145.t1 (E-value: 0.0; percent identity: 69.11% as determined by *blastp* ). The putative ortholog is flanked upstream by Augustus gene prediction JAEIGM010000023.g146.t1 and JAEIGM010000023.g147.t1, which correspond to *CF transmembrane conductance regulator* ( *Cftr* ) and *Coiled-coil domain containing protein 39* ( *Ccdc39* ) in *D. melanogaster* (E-value: 0.0 and 0.0 identity: 79.64% and 61.61%, respectively, as determined by *blastp* ; Figure 1A ; Altschul et al., 1990). The putative ortholog of *sad* is flanked downstream by Augustus gene prediction JAEIGM010000023.g144.t1 and GeMoMa gene predictions FBtr0082516_R0, FBtr0301042_R0, and FBtr82515_R0 (nested in FBtr0301042_R0, which correspond to *Cftr* , *CG6962* , *salsa* , and *mthl5* in *D. melanogaster* (E-value: 0.0 for all; identity: 64.05%, 66.19%, 86.34%, and 75.86%, respectively, as determined by *blastp* ). The putative ortholog assignment for *sad* in *D. cardini* is supported by the following evidence: The *tblastn* results are of good quality, and all coding sequences (CDS) and isoforms found in *D. melanogaster* also appear to be present in *D. cardini* . The gene predictions surrounding the *sad* ortholog are not fully conserved. *CG6962* and *salsa* with *mthl5* nested in it are downstream of *sad* as expected, but a copy of *Cftr* occurs between them and *sad* . Both of the upstream gene predictions, *Cftr* and *Ccdc39* , occur on chromosome 3R in *D. melanogaster* . However, *Cftr* is much further upstream and *Ccdc39* is downstream in *D. melanogaster* . This suggests the potential for multiple chromosomal inversions in this region. Inversions are common within the genus *Drosophila* and play an important role in speciation (Powell, 1997; Bhutkar et al., 2008; Reis et al., 2018). We conclude that the Augustus gene prediction JAEIGM010000023.g145.t1 is an ortholog of *sad* in *D. cardini* ( Figure 1A ).

***Protein Model -*** *sad* in *D. cardini* has 6 CDSs within the genome sequence. The only unique protein sequence (sad-PA) is translated from 1 mRNA isoform (sad-RA; Figure 1B ). Relative to the ortholog in *D. melanogaster* , the CDS number and protein isoform count are conserved *.* The sequence of sad-PA in *D. cardini* has 67.9% identity (81.0% similarity) with the protein-coding isoform sad-PA in *D. melanogaster* , as determined by *blastp* ( Figure 1C ). This level of divergence is not surprising given that *D. cardini* and *D. melanogaster* belong to two separate subgenera ( *Drosophila* and *Sophophora* respectively) that diverged approximately 45-60MYA (Russo et al., 1995; Tamura et al., 2004; Obbard et al., 2012). Coordinates of this curated gene model are archived in the CaltechDATA repository (see “Extended Data” section below).

## Methods

The annotation methods used in this project are adapted from those described in Rele et al. (2023), which includes algorithms, database versions, and citations for the complete annotation process developed for the Pathways Project. The methods for the current project are detailed in brief below with notes on significant differences between this protocol and the one described in Rele et al. (2023). The students use the GEP instance of the UCSC Genome Browser v.435 ( https://gander.wustl.edu ; Kent et al., 2002; Raney et al., 2024) to examine the genomic neighborhood of their reference detoxification gene in the *D. melanogaster* genome assembly (Aug. 2014; BDGP Release 6 + ISO1 MT/dm6). Students obtain the protein sequence for the *D. melanogaster* target gene for a given isoform and use a *tblastn* search of the sequence against their target *Drosophila* species genome assembly ( *D. cardini* (GCA_018903735.1 – Kim et al., 2021)) on the NCBI BLAST server ( https://blast.ncbi.nlm.nih.gov/Blast.cgi , Altschul et al., 1990) to identify the putative ortholog location. Students compare the genomic neighborhood of the putative ortholog to that of the reference gene in *D. melanogaster* . This local synteny analysis includes a minimum of two upstream and downstream genes relative to the potential ortholog. As no RefSeq protein data is available for these species, comparisons are based on gene predictions that correlate with gene expression data in the putative ortholog neighborhood. Using the multiple alignment tracks feature in the Genome Browser, students examine other sets of genomic evidence, including Spaln alignment of *D. melanogaster* proteins, multiple gene prediction tracks (e.g., GeMoMa, Augustus, NSCAN PASA-EST), and mixed sex adult RNA-Seq expression data from the target species generated by Erlenbach et al. (2023; https://doi.org/10.5061/dryad.hdr7sqvq2 ). Information on the genomic structure information (e.g., CDSs, intron-exon number, number of isoforms) for the reference gene in *D. melanogaster* is retrieved using Gene Record Finder ( https://gander.wustl.edu/~wilson/dmelgenerecord/index.html ; Rele et al *.,* 2023). To determine approximate splice sites within the target gene, a *tblastn* search using the CDSs from the *D. melanogaste* r reference gene against the putative ortholog location (10kb up- and downstream of the target gene prediction). Coordinates of the CDS(s) are refined by examining aligned RNA-Seq data, identifying canonical splice site sequences, and ensuring the maintenance of an open reading frame. Students confirm the biological validity of their target gene model using the FlySeq Gene Model Checker ( https://gander2.wustl.edu/~wilson/genechecker-flyseq/ ), which compares the hypothesized target gene model's structure and translated sequence against the *D. melanogaster* reference gene. At least two independent models for this gene are generated. These models are reconciled by a third independent researcher to produce the final model presented here. Note: comparison of 5' and 3' UTR sequence information is not included in this GEP CURE protocol.

## Data Availability

Description: Zipped archive containing FASTA, PEP, and GFF files for sad model. Resource Type: Model. DOI: https://doi.org/10.22002/0jmvb-n8f52
